# Discordance of diabetic retinopathy severity in a cohort of diabetic nephropathy patients: a cross-sectional case-control study in a new Mexican population of type 2 diabetes

**DOI:** 10.3389/fendo.2025.1638415

**Published:** 2025-08-01

**Authors:** Ashley Fitzgerald, Ryan Das, Cody J. Moezzi, Savannah R. Salazar, Rushi Mankad, Clifford R. Qualls, Andrea Cabrera, Ayushi Kathuria, Finny Monickaraj, Antonios Tzamaloukas, Arup Das

**Affiliations:** ^1^ Department of Ophthalmology and Visual Sciences, University of New Mexico School of Medicine, Albuquerque, NM, United States; ^2^ Ophthalmology Service, New Mexico Veterans Affairs (VA) Health Care System, Albuquerque, NM, United States

**Keywords:** diabetes mellitus, diabetic retinopathy (DR), proliferative diabetic retinopathy (PDR), glycated hemoglobin (HbA1c), diabetic nephropathy (DN), end stage renal disease

## Abstract

**Background:**

Although diabetic retinopathy (DR) and diabetic nephropathy (DN) are well known microvascular complications of diabetes, the correlation between DR and DN remains uncertain. Several studies have suggested differences in etiology and risk factors between these two complications.

**Objectives:**

To examine whether diabetic retinopathy (DR) and nephropathy (DN) have significant concordance in terms of severity progression in patients with type 2 diabetes.

**Methods:**

A case-control study was conducted involving two cohorts of type 2 diabetic patients from a New Mexican population. The cases had confirmed end-stage renal disease (ESRD; Stage 5, on dialysis, **e**GFR < 15 mL/min, n = 164), while the controls had mild diabetic nephropathy (DN) (Stage 1 or Stage 2, **e**GFR > 60 mL/min, n = 165). Systemic parameters were collected through retrospective chart reviews, which included HbA1c, blood pressure (BP), lipid levels, serum creatinine (Cr), and retinopathy status determined by dilated fundus examinations. Statistical analyses were conducted, encompassing univariate and multivariate logistic regression analyses for continuous variables, as well as a Chi-squared test for categorical variables.

**Results:**

The majority (65%) of the ESRD cohort had proliferative diabetic retinopathy (PDR), while 18% of patients exhibited no diabetic retinopathy (DR) or mild non-proliferative diabetic retinopathy (NPDR). Conversely, approximately 38% of the mild DN cohort had PDR. In the univariate analysis, ESRD was significantly associated with lower HbA1c levels (p<0.0001) and higher systolic blood pressure (p<0.0001). Within the ESRD cohort, the onset of PDR was significantly linked to younger age (p=0.0002), higher diastolic blood pressure (p=0.0319), and elevated LDL (p=0.0361). In the multivariate analysis, the development of PDR was inversely related to age (p=0.001, OR=0.95) and positively correlated with serum creatinine (p<0.0001, OR=1.25), systolic blood pressure (p=0.0221, OR=1.023), and albuminuria (p=0.0006, OR=4.65). HbA1c levels showed no significant correlation with the progression of PDR. The use of PDR as a screening tool for chronic kidney disease (CKD) has a sensitivity of 78.68% and a specificity of 51.16%, indicating that it is a suboptimal screening method.

**Conclusions:**

Our findings suggest discordance between the progression of diabetic retinopathy and nephropathy.

## Introduction

Diabetic retinopathy (DR), an important microvascular complication, affects about one-third of people with diabetes and is the leading cause of blindness globally (1). Similarly, diabetic nephropathy (DN), is a potential consequence of long-standing diabetes and the leading cause of chronic kidney disease (CKD) and end-stage renal disease (ESRD) across the world (2). Those with DR and CKD together had a 3.6-fold increased risk for all-cause mortality compared to people with diabetes with neither DR nor CKD (3). Prior research studies have implicated a significant clinical correlation between the two microvascular diseases (4), with large-scale clinical trials proposing endothelial dysfunction as a common pathogenic mechanism (5–7). However, some existing studies contradict this idea and found the presence of advanced nephropathy without concurrent retinopathy, suggesting differences in etiology and risk factors (8, 9).

Both the extent to which the many variables associated with microvascular diabetic disease affect disease progression and the correlation between DR and DN remains uncertain. Though the two diseases originate from endothelial dysfunction, a well-known phenomenon of long-standing diabetes, DR status is suboptimal in predicting DN, with a sensitivity of 0.64 and specificity of 0.77 (10, 11). Discordance between DR and DN has been observed in specific patient populations. Cases of advanced clinical retinopathy but no signs of end-stage renal disease, with minimal albuminuria and normal glomerular structure, have been documented (12). Conversely, studies have found patients with advanced diabetic nephropathy and little to no retinopathy, deeming them extreme phenotypes (13).

Several other factors apart from diabetes can also cause CKD. In a person with severe albuminuria, the presence of DR is a strong indicator of CKD due to diabetes. In contrast, in a person with minimal or moderate albuminuria, the absence of DR suggests other nondiabetic causes of CKD like hypertension, renal artery stenosis, or other systemic diseases (14–16). Thus, DR examination in a patient with diabetes-related CKD provides an additional tool to investigate the etiology of the CKD, as kidney biopsy is an invasive procedure and is not always feasible.

Our study aimed to identify risk factors that contribute to the development of end-stage renal disease and sight-threatening complications of DR in a unique New Mexican cohort of patients with type 2 diabetes. Additionally, we wanted to determine the associations between the occurrence of two microvascular complications, DN and DR. Our results may have clinical applications in predicting the severity of nephropathy based on retinopathy status.

## Materials and methods

This study was performed per the Helsinki Declaration of 1975, and the study protocol was approved by the Institutional Review Board of the University of New Mexico Health Sciences Center (UNMHSC) (HRRC 20-612). This retrospective, case-control study was designed to determine positively associated risk factors in patients with confirmed type 2 diabetes and secondary microvascular complications between October 2016 and December 2021. The necessity for obtaining consent forms was exempted as the study was based on a retrospective chart analysis of aggregated data from deidentified information. No protected information was accessed, and there was no intervention therapy conducted in this study.

### Subjects

Retrospective patient data was obtained via the Cerner PowerChart patient charting system utilized by the UNMHSC. To identify these cases, initially the diagnostic codes were used from the International Classification of Diseases, 10^th^ Edition (ICD-10; ICD10Data.com) and all patients diagnosed with DN (diabetes type 2 with diabetic nephropathy), ESRD, mild NPDR (nonproliferative diabetic retinopathy) and PDR (proliferative diabetic retinopathy) were identified. After final review, a total of 329 patients were used in this study.

### Retinopathy assessment

Each patient case was reviewed further to ensure the ICD-10 code diagnosis matched phenotype physical findings. Any cases where phenotypic physical exam findings and ICD-10 were incongruent were excluded. Ocular exams, including best-corrected visual acuity, stereoscopic biomicroscopy, and indirect ophthalmoscopy, were performed in all patients. Fundus photographs (Wide angle OPTOS) were obtained in 74% of subjects, and optical coherence tomography images (Heidelberg Spectralis) were done in 100%. Phenotypes included in this study were diabetes with no retinopathy, mild NPDR, and PDR. To confirm mild NPDR, patients must have had a few microaneurysms on the fundus exam. PDR verification was done via chart review of the patient history of fundus imaging demonstrating neovascularization, pan-retinal photocoagulation (PRP) laser treatment, or pars plana vitrectomy (PPV). The phenotype of each case was confirmed by the study team’s senior retina specialist (AD) based on clinical notes and examination drawings, fundus photographs, and OCT images.

### Nephropathy assessment

All ESRD nephropathy cases were identified using ICD-10 codes determined by previous dialysis treatment. Then, each patient’s case was further reviewed to ensure that the ICD-10 code diagnosis was compatible with the physical findings of the phenotype and lab values. The estimated glomerular filtration rate (**e**GFR, mL/min), calculated from the Chronic Kidney Disease Epidemiology Collaboration Equation (CKD-EPI) (17), was collected, and the most recent value was recorded. Staging was determined using the National Kidney Foundation guidelines: Stage 1 CKD (Mild): eGFR ≥90; Stage 2 CKD: 60-90; Stage 3 CKD: 30-59; Stage 4 CKD: 15-29; Stage 5 CKD: <15. Each ESRD case was reviewed, and patients with stage 5 CKD (eGFR <15 mL/min) only were included. Patients in the control group included CKD stages 1 through 2 (eGFR >60 mL/min) (Mild nephropathy). The patients’ most recent urine albumin-to-creatinine ratio (UACR, mg albumin/g creatinine) was recorded to assess albuminuria. Albuminuria was classified according to the National Kidney Foundations’ set values: normoalbuminuria (≤30mg/g), microalbuminuria (30 < UACR ≤ 300 mg/g), or macroalbuminuria (>300 mg/g) (14).

### Systemic factors

Systemic factors collected included both modifiable and non-modifiable risk factors. These factors were selected based on a literature review examining DR and DN progression and associated systemic risk factors.

Non-modifiable risk factors assessed were age, sex, and race/ethnicity, retrieved from patient demographical information during initial visits. Race/ethnicity was categorized as non-Hispanic White, Hispanic, Black, Asian, Others (American Indian), or Decline to Specify. A diagnosis of type 2 diabetes was confirmed from the patient’s primary care physician’s notes, glycated hemoglobin (HbA1c) levels, and the patient’s medications. All patients had a duration of diabetes of at least 10 years, as some form of retinopathy is usually present after this duration. Past HbA1c values were collected and averaged over 10 years. Duration of diabetes was determined via chart review and patient report of diagnosis year. We reviewed patients’ active medication lists from their last visit to determine their use of any insulin, angiotensin-converting enzyme (ACE) inhibitors, calcium channel blockers, and beta blockers. Body mass index (BMI, kg/m^2^) was autogenerated via Cerner PowerChart using patients’ most recent recording of height and weight. Systolic blood pressure (SBP), diastolic blood pressure (DBP), and fasting lipid levels (total cholesterol, LDL, and triglyceride) were averaged to calculate mean values over 2 years. History of dialysis, smoking, myocardial infarction, stroke, and cancer was determined via chart review and/or patient report during initial visits.

### Statistical analysis

All statistical tests were run using the SAS program, version 9.4. P-values of < 0.05 were considered significant. Univariate and multivariate logistic regression analyses were performed for each outcome and PDR and ESRD progression. The backward and stepwise selection was used in the multivariate analysis and confirmed using the forward selection model for each outcome. The goodness of fit statistics was assessed for each outcome. Each outcome was analyzed separately, and baseline means and proportions were compared using two sample student’s t-tests or chi-squared tests. All values that were averaged were done so over two years. The diagnostic value of using PDR to predict ESRD was assessed by sensitivity, specificity, positive likelihood ratio (PLR), negative likelihood ratio (NLR), confidence intervals (CI), odds ratio (OR), and area under the receiver operating characteristic curve (AUC/ROC). By determining the sensitivity and specificity of retinopathy status in determining nephropathy status, we aimed to understand the clinical use and potential value of assessing retinopathy status in predicting the severity of diabetic nephropathy. While sensitivity gives information for ruling out a disease, specificity gives information on ruling out the disease. Sensitivity and specificity are inversely proportional, meaning that as one increases, the other decreases (18). The ROC curve was calculated based on the true/false positive and true/false negative values. Aninterpretative scale for AUC values includes that an AUC 0.5 is no better at predicting disease than by chance alone. The maximum AUC of 1 means the test perfectly differentiates disease and non-disease outcomes. An AUC of 0.7 to 0.8 is deemed to predict disease state reasonably. The closer the AUC is to 1, the better it is at predicting disease state.

Albuminuria was initially analyzed as a trinary variable: macro-, micro-, and normo-albuminuria. However, there was no statistically significant difference between macro- and micro-albuminuria, so we combined the two into one category. Consequently, combined albuminuria with macro- and micro- was analyzed and compared to normoalbuminuric patients. It is also important to note we examined HbA1c as both a continuous and a categorical variable. HbA1c categories were defined as follows: 1) < 7% good control, 2) 7-9% intermediate control, and 3) > 9% poor control (19).

## Results

For our study, 164 patients met the criteria for ESRD (cases), and 165 patients for mild nephropathy (controls). Of this ESRD cohort, 65% of the patients (n=107) had advanced retinopathy as PDR. However, a sizeable amount, 18% (n=29) had mild eye disease (mild NPDR or no retinopathy). Of our mild nephropathy control group (n = 165), only 40% (n=66) patients had a mild eye disease, while a significant number of patients (39%; n=64) developed PDR. We included only the extreme ends of the DR phenotype curve, mild NPDR *vs*. advanced retinopathy (PDR) and excluded all moderate eye diseases (moderate/severe NPDR) from our statistical analysis.

### Univariate analysis

We compared the baseline characteristics of those with ESRD to those with mild nephropathy (**Table 1**). Several factors were significantly associated with ESRD compared to mild nephropathy, regardless of retinopathy status. These included duration of diabetes (p=0.0369), systolic blood pressure (p<0.0001), serum creatinine (p<0.0001), urine protein (p=0.0224), urine total protein (p=0.0129), and urine albumin/Cr (p<0.0001). The mild nephropathy group had a significantly higher proportion of females (p=0.0046). ACE-inhibitor use (p<0.0001), calcium channel blocker use (p<0.0001), and beta-blocker use (p<0.0001) were all significantly associated with the ESRD group. HbA1c was inversely associated with ESRD (p<0.0001), with the ESRD group showing a lower mean HbA1c (7.75%) compared to the mild nephropathy group (8.78%). This difference may be attributed to dialysis or better clinical regulation of glycemic control in ESRD patients. As expected, ESRD was inversely associated with GFR (p<0.0001), Hb (p<0.0001), and Hct (p<0.0001).

**Table 1:** Baseline characteristics of mild nephropathy (controls) and ESRD (cases) cohorts.

Characteristics	Mild Nephropathy	ESRD	P Value
	Mean (SD)	Mean (SD)	
Age (years)	63.08 (13.08)	62 (11.03)	0.67
HbA1c (%)	8.78 (1.70)	7.75 (1.80)	<0.0001
Duration of DM (years)	21.53 (9.17)	24.29 (10.42)	0.037
BMI (kg/m2)	30.70 (6.92)	29.63 (7.36)	0.22
SBP (mmHg)	133.6 (12.92)	143.2 (19.32)	<0.0001
DBP (mmHg)	71.10 (7.24)	72.62	0.17
Total Cholesterol (g/dL)	152.5 (42.00)	156.6 (49.12)	0.5
LDL (g/dL)	76.42 (35.18)	77.7 (45.00)	0.81
HDL (g/dL)	45.55 (13.38)	44.35 (15.64)	0.53
Triglycerides (g/dL)	159.4 (101.2)	181 (137.4)	0.18
GFR (mL/min/1.73m^2^)	80.70 (15.04)	17.25 (19.21)	<.0001
Cr (mg/dL)	0.89 (0.21)	5.07 (2.18)	<0.0001
Duration of ESRD (years)	/	4.72 (2.76)	/
Urine Cr (mg/dL)	101.7 (57.49)	89.70 (44.59)	0.09
Urine Protein (mg/dL)	1.19 (3.00)	7.48 (24.37)	0.02
Urine Total Protein (mg/dL)	59.35 (73.61)	505.0 (1663.1)	0.01
Urine Albumin Cr (mg/g)	328.5 (1053.6)	2834.5 (2466.8)	<0.0001
Hemoglobin (g/dL)	13.61 (3.03)	10.77 (1.80)	<0.0001
Hematocrit (%)	41.69 (6.91)	35.84 (26.13)	0.01
	n (% of total)	n (% of total)	
Females	70 (54.26)	50 (36.76)	
Males	59 (45.74)	86 (63.24)	
Race by ethnicity			
Asian	7 (6.31)	4 (2.94)	
Black	1 (0.9)	5 (3.68)	
Hispanic	40 (36.04)	48 (35.29)	
White	56 (50.45)	56 (41.18)	
Other (AI)	6 (5.41)	11 (8.09)	
Albuminuria			<0.0001
Macroalbuminuria	14 (13.46)	29 (30.53)	
Microalbuminuria	48 (46.15)	53 (55.79)	
Normoalbuminuria	42 (40.38)	13 (13.68)	
History of dialysis	1 (0.78)	132 (97.06)	<0.0001
Use of insulin	96 (74.42)	101 (74.81)	1
Use of ACE inhibitor	63 (48.84)	21 (15.44)	<0.0001
Use of calcium channel blocker	36 (28.13)	76 (55.88)	<0.0001
Use of beta blocker	35 (27.34)	88 (64.71)	<0.0001
Use of EPO	0	12 (8.82)	0.0004

Means of univariate non-binary variables comparing Mild Nephropathy vs. ESRD. ESRD, End stage renal disease; Cr, Creatinine; ACE, Angiotensin converting enzyme; EPO, Erythropoietin.

In our univariate logistic regression analysis, we determined which variables were significantly associated with both retinopathy status and nephropathy status. In the ESRD group, the factors that were significantly associated with PDR (**Table 2**) included diastolic blood pressure (p=0.031), low-density lipoprotein (LDL) (p=0.036), serum creatinine (Cr) (p=0.012), and ACE inhibitor use (p=0.007). Two factors inversely associated included age (p=0.0002) (mean age of 61 yrs. in the PDR group *vs*. 70 yrs. in mild retinopathy) and GFR (p=0.002). Interestingly, HbA1c (p=0.506) was not significantly associated with PDR development (mean HbA1c level 7.81 in the PDR group *vs*. 7.54 in mild retinopathy). Duration of diabetes was not significantly associated with PDR development.

**Table 2:** Means of univariate non-binary variables comparing Mild NPDR *vs*. PDR in the ESRD as well as mild nephropathy cohorts.

Variable	ESRD cohort – Mild NPDR *vs* PDR			Mild Nephropathy cohort – Mild NPDR *vs* PDR		
	Mild NPDR	PDR	P Value	Mild NPDR	PDR	P Value
	Mean (SD)	Mean (SD)		Mean (SD)	Mean (SD)	
Age (years)	69.7 (10.13)	61.0 (10.56)	0.0002	65.86 (13.00)	60.92 (12.78)	0.03
HbA1c (%)	7.54 (1.87)	7.80 (1.78)	0.5	8.39 (1.52)	9.18 (1.79)	0.0094
Duration of DM (years)	26.47 (14.63)	23.68 (8.94)	0.39	20.16 (9.27)	23.12 (8.88)	0.0778
BMI (kg/m2)	29.56 (1.60)	29.65 (0.68)	0.95	29.82 (6.28)	31.67 (7.49)	0.14
SBP (mm Hg)	137.2 (19.32)	144.8 (19.09)	0.06	131.2 (13.77)	136.2 (13.73)	0.04
DBP (mm Hg)	69.07 (9.51)	73.59 (10.63)	0.03	69.49 (6.06)	72.85 (8.02)	0.0098
Total cholesterol (g/dL)	142.2 (33.10)	160.1 (52.8)	0.08	150.6 (38.71)	155.0 (46.42)	0.6
LDL (g/dL)	65.69 (26.99)	81.51 (48.88)	0.03	73.80 (30.28)	79.99 (41.00)	0.38
HDL (g/dL)	45.16 (10.86)	44.10 (16.92)	0.7	46.87 (13.74)	43.75 (12.81)	0.22
Triglycerides (g/dL)	165.7 (100.8)	185.6 (146.8)	0.43	154.0 (94.70)	166.6 (109.6)	0.52
GFR (mL/min/1.73m^2^)	30.62 (26.52)	13.59 (14.86)	0.0022	82.32 (15.37)	79.02 (14.62)	0.2134
Cr (mg/dL)	3.70 (3.33)	5.45 (2.55)	0.01	0.86 (0.18)	0.91 (0.23)	0.21
Duration of ESRD (years)	5.5 (3.64)	4.52 (2.27)	0.22	/	/	/
Urine Cr (mg/dL)	93.68 (39.06)	88.47 (46.35)	0.59	106.5 (57.19)	95.45 (57.88)	0.31
Urine Protein (mg/dL)	2.52 (3.03)	8.81 (27.30)	0.06	0.54 (0.42)	1.59 (3.80)	0.29
Urine total protein (mg/dL)	173.5 (171.0)	599.8 (1875.7)	0.06	55.65 (77.58)	62.86 (71.36)	0.75
Urine albumin Cr (mg/g)	1972.5 (1954.5)	3059.3 (2554.2)	0.12	105.1 (206.6)	612.7 (1534.1)	0.34
Hemoglobin (g/dL)	10.92 (2.02)	10.73 (1.75)	0.64	13.88 (3.72)	13.36 (2.18)	0.36
Hematocrit (%)	34.23 (5.71)	36.27 (29.32)	0.5	42.45 (7.94)	40.97 (5.74)	0.24
	n (% of total)	n (% of total)		n (% of total)	n (% of total)	
Females	15 (51.72)	35 (32.71)		33 (50)	37 (58.73)	
Males	14 (48.28)	72 (67.29)		33 (50)	26 (41.27)	
Race by ethnicity			0.02			<0.0001
Asian	2(6.9)	2 (1.87)		7 (11.86)	0	
Black	3(10.34)	2 (1.87)		1 (1.69)	0	
Hispanic	8(27.59)	40 (37.38)		10 (16.95)	30 (57.69)	
White	15(51.72)	41 (38.32)		38 (64.41)	18 (34.62)	
Other (AI)	0	11 (10.28)		3 (5.08)	3 (5.77)	
Albuminuria			0.0039			<0.0001
Macroalbuminuria	2 (9.52)	27 (36.49)		7 (11.86)	7 (15.56)	
Microalbuminuria	12 (57.14)	41 (55.41)		18 (30.51)	30 (66.67)	
Normoalbuminuria	7 (33.33)	6 (8.11)		34 (57.63)	8 (17.78)	
Use of insulin	21 (15.56)	80 (59.26)	0.81	42 (63.64)	54 (85.71)	0.0048
Use of ACE inhibitor	0	21 (19.63)	0.0072	33 (50)	30 (41.62)	0.8607
Use of calcium channel blocker	12 (41.38)	64 (59.810	0.09	17 (26.15)	19 (30.16)	0.6955
Use of beta blocker	20 (68.97)	68 (63.55)	0.66	21 (32.31)	14 (22.22)	0.2366
Use of EPO	3 (10.34)	9 (8.41)	0.71	/	/	/

In the mild nephropathy group, factors that were significantly associated with PDR compared to mild retinopathy included systolic (p=0.044) and diastolic blood pressure (p=0.009), urine albumin/CR (p=0.034), HbA1c (p=0.0094), and insulin use (p=0.0048) (**Table 2**). The only factor that was inversely associated with PDR included age (p=0.031; mean age of 61 yrs. in the PDR group *vs*. 66 yrs. in the mild retinopathy group). Similar to the ESRD group, the duration of diabetes was not significantly associated with PDR.

When comparing all subjects with PDR to all with mild retinopathy, irrespective of nephropathy status (**Table 3**), several factors were significantly associated with PDR. These included systolic (p<0.0001) and diastolic blood pressure (p=0.0002), serum Cr (p<0.0001), urine protein (p=0.0450), urine total protein (p=0.0421), and urine albumin/Cr (p<0.0001). Again, the factors associated inversely with PDR included age (p=0.0001), GFR (p<0.0001), and Hb (p=0.0047). Notably, HbA1c was not significantly associated with PDR (p=0.4165).

**Table 3:** Means of univariate non-binary variables comparing Mild NPDR *vs*. PDR in the combined mild nephropathy and ESRD cohorts.

Variable	Combined ESRD/mild nephropathy cohort-PDR *vs*. mild retinopathy		
	Mild NPDR	PDR	P Value
	Mean (SD)	Mean (SD)	
Age (years)	67.03 (12.27	60.94 (11.39)	0.0001
HbA1c (%)	8.13 (1.67)	8.32 (1.90)	0.41
Duration of DM (years)	21.83 (11.20)	23.45 (8.88)	0.2534
BMI (kg/m2)	29.74 (6.97)	30.37 (7.27)	0.49
SBP (mmHg)	133.1 (15.83)	141.7 (17.79)	<0.0001
DBP (mmHg)	69.36 (7.25)	73.32 (9.75)	0.0002
Total cholesterol (g/dL)	149.0 (37.04)	158.3 (50.54)	0.11
LDL (g/dL)	71.39 (29.43)	80.97 (46.07)	0.05
HDL (g/dL)	46.36 (12.91)	43.98 (15.54)	0.21
Triglycerides (g/dL)	157.3 (96.07)	178.7 (134.4)	0.16
GFR (mL/min/1.73m^2^)	66.54 (30.75)	37.98 (34.98)	<.0001
Cr (mg/dL)	1.73 (2.24)	3.75 (2.98)	<0.0001
Duration of ESD (years)	5.50 (3.64)	4.52 (2.47)	0.22
Urine Cr (mg/dL)	103.1 (52.99)	91.21 (51.07)	0.11
Urine Protein (mg/dL)	1.81 (2.60)	7.42 (24.71)	0.04
Urine total protein (mg/dL)	114.6 (144.0)	475.9 (1658.4)	0.04
Urine albumin Cr (mg/g)	434.7 (1084.7)	1863.2 (2438.9)	<0.001
Hemoglobin (g/dL)	12.89 (3.53)	11.69 (2.29)	0.0047
Hematocrit (%)	39.71 (8.22)	37.98 (23.72)	0.39
	n (% of total)	n (% of total)	
Females	48 (50.53)	72 (42.35)	
Males	47 (49.47)	98 (57.65)	
Race by ethnicity			<0.0001
Asian	9 (10.23)	2 (1.26)	
Black	4 (4.55)	2 (1.26)	
Hispanic	18 (20.45)	70 (44.03)	
White	53 (60.23)	59 (37.11)	
Other (AI)	3 (3.41)	14 (8.81)	
Albuminuria	9 (11.25)	34 (28.57)	<0.0001
Macroaalbuminuria	30 (37.5)	71 (59.66)	
Microalbuminuria	41 (51.25)	14 (11.7)	
Normoalbuminuria	28 (29.47)	105 (61.76)	<0.0001
History of dialysis	41 (43.62)	60 (35.5)	0.23
Use of insulin	63 (66.32)	134 (79.29)	
Use of ACE inhibitor	33 (34.74)	51 (30)	
Use of calcium channel blocker	29 (30.85)	83 (48.82)	
Use of beta blocker	41 (43.62)	82 (48.24)	

### Multivariate analysis

**Figure 1:**
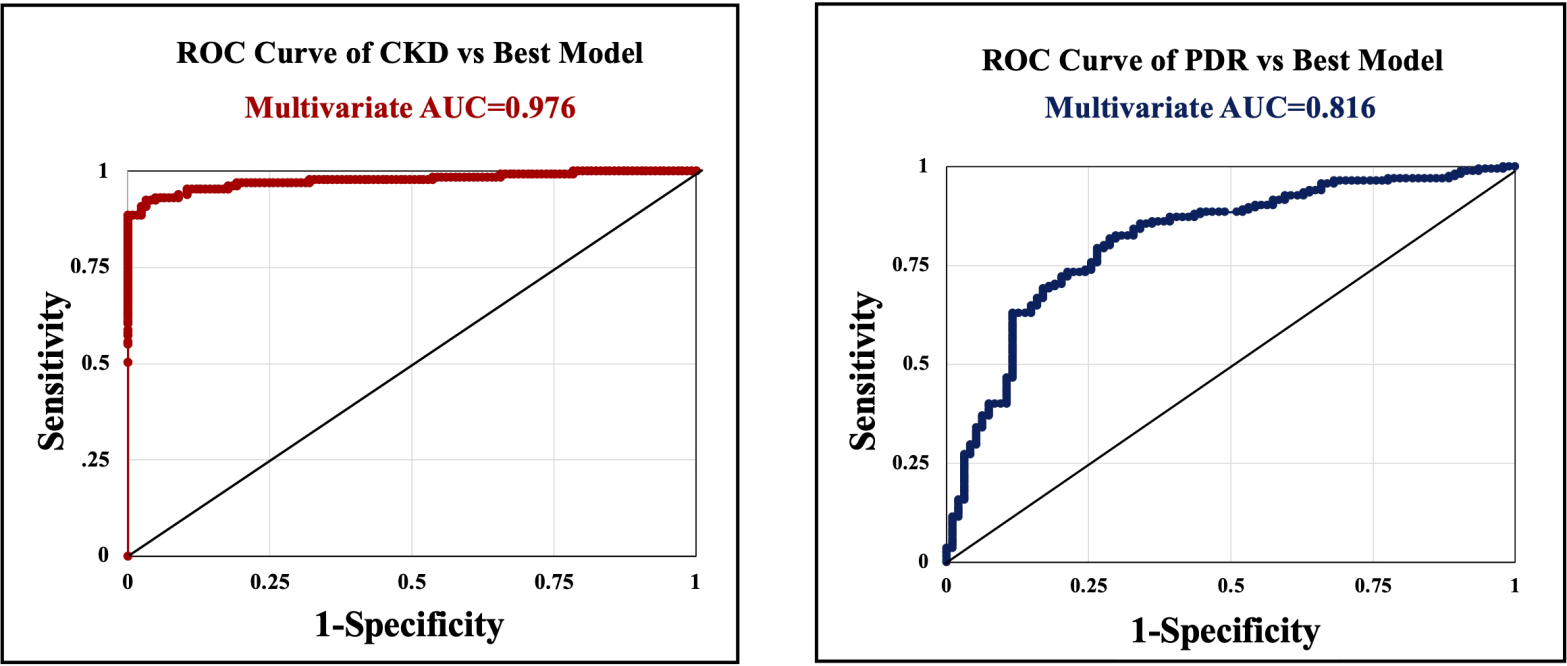
Receiver operating characteristic (ROC) curves with calculated area under the curve (AUC) statistics. Serum creatinine that was significantly associated with ESRD in the multivariate analysis, was an excellent predictor (AUC=0.976)(left). Risk factors that were significantly associated with PDR included albuminuria, serum creatinine level, and systolic blood pressure, also demonstrated strong predictive power (AUC=0.816)(right). However, PDR was not significantly associated with ESRD in the multivariate analysis when adjusted for HbA1c and Cr.

In our multivariate analysis, risk factors that were significantly associated with PDR included albuminuria (p=0.0006, OR=4.65), serum creatinine level (p<0.0001, OR=1.345), and systolic blood pressure (p=0.0221, OR=1.025) (**Table 4**). These variables demonstrated strong predictive power (AUC=0.816) (**Figure 1**). Race and ethnicity significantly predicted PDR (p<0.002). However, when individual races were analyzed, Hispanics were significantly associated (p=0.0204, OR=3.245) with PDR. Age was a protective factor associated with PDR (p=0.0027, OR=0.96). HbA1c was not significantly associated with PDR when adjusted for higher frequency variables (p=0.6).

**Table 4:** Multivariate analysis results comparing either PDR *vs*. mild NPDR (left) or ESRD *vs*. mild DN (right).

*PDR vs. mild Retinopathy*			*ESRD vs. mild Nephropathy*		
Variable	*OR (CI)*	*P Value*	*Variable*	*OR (CI)*	*P Value*
Age (years)	0.95 (0.92, 0.98)	0.001	Age (years)	–	0.64
HbA_1c_ (%)	1.06 (0.92, 1.22)	0.43	HbA_1c_ (%)	0.52 (0.32, 0.85)	0.008
SBP (mmHg)	1.02 (1.00, 1.04)	0.02	BMI (Kg/m^2^)	–	0.51
Serum cr (mg/dL)	1.345	<0.0001	SBP (mmHg)	–	0.67
Albuminuria	4.65	0.0006	Serum Cr (mg/dL)	285.03 (28.77, >999.99)	<0.0001
Race by ethnicity *vs* White			Hemoglobin (hgb)	0.94 (0.74, 1.25)	0.61
Asian	0.20 (0.04, 0.97)	0.095	Hematocrit (hct)	–	0.41
Black	0.45 (0.08, 2.55)	0.10			
Hispanic	3.5 (1.85, 6.61)	0.003			
Other (AI)	10.78 (1.37, 85.72)	0.055			

Only significant variables listed.

Variables that were significantly associated with ESRD in the multivariate analysis included serum Cr (p<0.0001, OR=285.034), which was an excellent predictor (AUC=0.976). HbA1c was inversely associated with ESRD (p=0.0088, OR=0.516). PDR was not significantly associated with ESRD in the multivariate analysis when adjusted for HbA1c and Cr.

### Sensitivity and specificity: a screening tool for diabetic nephropathy

In our study, the sensitivity of PDR predicting ESRD was 79% while the specificity was 51%, which are generally low values for an effective screening tool.

## Discussion

In our case-control study of a New Mexican population of type 2 diabetes, we have identified several significant risk factors that show the relationship between the progression of diabetic etinopathy and diabetic nephropathy. A substantial number of patients (18%) in the ESRD cohort showed no eye disease or mild eye disease or mild NPDR; conversely, the mild DN cohort had a significant number of patients (39%) with advanced eye disease, PDR. Such a discrepancy in DR status in both nephropathy cohorts demonstrates a significant discordance between these two microvascular complications of diabetes. Our multivariate analysis revealed the strongest risk factors for developing PDR: albuminuria, higher systolic blood pressure, serum creatinine, and younger age. One of the important findings of our study was that the emergence of PDR was not significantly associated with glycemic control (HbA1c levels) in the univariate analysis of the ESRD cohort, the combined ESRD/mild DN cohorts, or even in the multivariate analysis. Our use of ROC curves demonstrated that PDR has poor predictability and is not a sufficient screening tool for determining the nephropathy status. We speculate that “other factors” beyond glycemic control, like inherent genetic differences, may play a component in the difference in progression rate for these two important complications of diabetes.

Of note, in the univariate analysis of the ESRD cohort, HbA1c was inversely associated with the development of ESRD, with the ESRD group showing lower average HbA1c levels than the mild nephropathy group. This was contradictory to studies that found that higher thresholds of HbA1c (above 7%) were associated with both microvascular diseases (20). However, other studies propose that individual variability or change in HbA1c is more significantly associated with diabetic nephropathy than mean HbA1c levels (21). We postulate that our study’s findings could be due to individual variability in HbA1c as described in previous studies, the effect of dialysis on HbA1c (22), and perhaps more stringent regulation of HbA1c with regular checkups, as disease severity requires more consistent monitoring of glycemic control. As with our PDR univariate analysis, we found blood pressure and serum creatinine to be significantly associated with ESRD. Variables that extend significance to both microvascular diseases may be key in predicting clinical outcomes.

Our study also revealed a highly significant association of high LDL levels with PDR (p =0.036). Studies have shown oxidized low-density lipoprotein to be associated with the incidence of proliferative retinopathy and other complications of type 1 diabetes mellitus. A recent study on Swedish children and young adults with type 1 diabetes showed that those with higher LDL cholesterol level have an increased risk of retinopathy and nephropathy independent of glycemia (23). The Wisconsin Epidemiological Study showed an increased risk of incident PDR with an increase in oxidized LDL level (24).

In the multivariate analysis, the most strongly associated PDR variables included serum creatinine, systolic blood pressure, and albuminuria. Previous studies have found that serum creatinine is positively associated with the development and severity of diabetic retinopathy (25). Similarly, systolic blood pressure has also been correlated with advanced retinopathy (26). Albuminuria is a surrogate marker for the progression and detection of diabetic nephropathy, and a more significant stage of albuminuria correlated to nephropathy progression is associated with the prevalence of diabetic retinopathy. However, these studies note the importance of predictive value with other known factors (27, 28). Established clinical values and cutoffs for these variables may prove helpful in predicting retinopathy status and guiding specialist referrals. When analyzing the races and ethnicities, Hispanics were found to be significantly associated with PDR compared to other ethnic backgrounds. This confirms prior evidence and research that consider Hispanics and Latinos to be at higher risk for advanced retinopathy (27, 29).

Variables significantly associated with ESRD in our multivariate analysis included serum creatinine. This finding was expected as serum creatinine levels increase as the filtration system of the kidney deteriorates with the progression of diabetic nephropathy. Serum creatinine is an established risk factor for diabetic kidney disease and may be helpful when considering other known risk factors in order to predict the progression and severity of the disease (30). We found that HbA1c was inversely associated with ESRD in our multivariate analysis, as we had seen in our univariate analysis. As mentioned with the univariate results, we hypothesize that this could be due to several factors, including dialysis impacting glycemic control, a and perhaps better control of HbA1c with more severe nephropathy due to more regular monitoring by providers.

Strong correlations between the development of diabetic nephropathy and genetics have been found, with single nucleotide polymorphisms (SNPs) and other genetic markers being associated (31). Similarly, studies have associated genetic factors with the development of diabetic retinopathy in several different populations (32–34). These findings suggest that genetics can have an underlying protective or deleterious effect on the development of DN and DR. While the two microvascular diseases share similar implicated etiologies, pathways, and progression, individual patient genetic makeup could significantly impact the overall outcome. Additionally, variables such as HbA1c positively correlate with DR and DN (29). However, the predictive value of HbA1c has been questioned in some studies, with a meta-analysis even contradicting its positive association with diabetic retinopathy (35). Other studies have predicted that glycemic control can only explain about 11% of the risk for the progression of retinopathy to proliferative diabetic retinopathy (PDR) (36). The additive effect of other contributing variables, such as cholesterol, blood pressure (BP), and serum and urine lab values in developing DR/DN remains largely unclear. Some populations may express protective factors that delay the progression of both DR and DN. Conversely, some ethnic and racial groups have been found to have a predisposition to DR and DN; for example, higher prevalence rates have been observed in Latino populations (37, 38). Furthermore, a genome-wide association study that accounted for glycemic control revealed higher rates of nephropathy in African American, Mexican, and American Indian populations compared to European ancestry counterparts (39).

An important question that has clinical relevance is if the presence of diabetic retinopathy is an accurate tool in diagnosing the incidence and severity of diabetic nephropathy. While kidney biopsy remains the gold standard for diagnosing and staging diabetic nephropathy, less invasive methods are available. According to the American Diabetes Association, urine albumin excretion, urine albumin to creatinine ratio, and estimated glomerular filtration rate (eGFR) through a calculation that includes serum creatinine, age, and weight of the patient can also be used. Due to the invasive nature of renal biopsy, DR status, both alone and in conjunction with lab values, is also being increasingly utilized in diagnosing DN (40). Although some studies have proposed that DR is an accurate predictor of DN (41), others have noted important limitations to consider. As discussed earlier, studies have found low concordance rates between the diseases and have observed extreme phenotypes that exist where patients exhibit either DN or DR, but not both concurrently. Other studies have found no significant association between diabetic retinopathy and end-stage renal disease (42). In addition, studies have noted the importance of identifying non-diabetic renal disease in diabetic patients (NDRD) in relation to diabetic retinopathy (43). Without renal biopsy, DN and NDRD are often difficult to differentiate based on lab values alone, so the presence of DR is often used to diagnose DN (44). Patients with diabetic retinopathy and abnormal lab values associated with kidney dysfunction are often diagnosed with DN without biopsy, but biopsy studies have shown that a small percentage (<10%) of these patients did in fact have NDRD (43). Furthermore, diabetic patients misdiagnosed with DN rather than NDRD may confound the results of studies that explore the relationship between DR and DN (44). Given these variable findings, we aimed to determine the predictive ability of DR in diagnosing DN through measuring sensitivity and specificity.

Whether the discordance between DR and DN is due to structural differences between these capillaries of two target organs is unclear. The glomerular endothelium has fenestrations that facilitate ultrafiltration into the Bowman’s capsule similar to the choroidal capillaries, whereas the retinal capillaries have ultrastructural differences as they have tight junctions between endothelial cells forming the inner blood-retinal barrier (BRB) (45). Also, the kidney is wrapped in a capsule that helps limit renal swelling with its resistance to stretching, whereas the retina can freely expand on the vitreous side with increased thickness with the alteration of the BRB in diabetic retinopathy. A recent spatial transcriptomic study on microvascular endothelial cells (MECs) from diabetic and non-diabetic mouse models showed that MECs from the retinas are distinct from the kidneys both at the basal state and in their responses to hyperglycemia in diabetes (46).

Sensitivity and specificity are important metrics for assessing screening ability. As a screening tool, sensitivity can be used to rule out diseases, while specificity can be confirmatory in ruling in a disease. In our analysis, advanced retinopathy alone had a sensitivity of 79% concordance with respect to ESRD and a specificity of 51%. While there are no set gold standards for percentage cutoffs of sensitivity and specificity because these accepted percentages are variable with severity of disease, in general, greater than 90% is considered good to excellent, while between 80% and 90% is considered moderate. Therefore, because diabetic nephropathy is a severe complication of type 2 diabetes, we consider advanced retinopathy to be a poor screening tool for advanced nephropathy. This has clinical relevance because it is expected that patients undergo screening for other diabetic complications if they have already progressed to PDR, however it may not be assumed that they have advanced nephropathy simply by retinopathy evaluation.

Interestingly, the relationship between DN and DR in type 2 diabetics is less direct than in type 1 diabetics, as diabetic retinopathy usually emerges before diabetic nephropathy in type 1 diabetics (47). In addition, diabetic nephropathy without concurrent diabetic retinopathy is much less frequent in type 1 diabetics compared to type 2 diabetics (48). This is supported by findings from the Renal Insufficiency And Cardiovascular Events (RIACE) Italian multicenter study, which also concluded that concordance between CKD and DR is low in type 2 diabetics compared to type 1 diabetics (49).

Limitations of our study include its retrospective design, which prevents this study from drawing a direct cause-effect relationship between risk factors and retinopathy or nephropathy phenotype. Also, there was missing information from some patients’ charts, and some risk factors we evaluated had low response rates. This creates a potential selection bias. To alleviate this concern, we excluded any variables with more than 25% of data missing from our final analysis. Information was collected retrospectively with a 5-year date range and criteria outlined in our methods section. Unfortunately, not all patients had regular follow-ups, and some had intervals of time that could not be accounted for. This could lead to an underestimation of the true risk factors in DR and DN. We utilized a 2-year average of most of our risk factors to better correct for lapses in data. Ethnicity or race was self-identified by patients, who refused to answer as an option, therefore leading to variability in response and a possible cause of lack of statistical significance in some of our analyses.

In summary, both diabetic retinopathy and diabetic nephropathy have known modifiable risk factors. The assessment of such diseases should be based on clinical expertise and measurement of quantitative and qualitative variables. Glycemic control alone is not a reliable way to predict the severity of these two microvascular diseases. Progression or severity of retinopathy is not always concurrent with nephropathy, and each should be evaluated separately. The reason for disjunction in concordance may be due to individual genetic makeup. We hypothesize that some genetic factors are protective while others predispose individuals to advanced or faster disease progression. Our study may be helpful clinically to better weigh risk factors to predict disease, intervention, and lifestyle modifications for targeted variables. As the study’s retrospective design can only infer associations rather than establish causality, further carefully designed observational studies are warranted to explore this association.

## Data Availability

The raw data supporting the conclusions of this article will be made available by the authors, without undue reservation.
